# Janus kinase and calcineurin‐inhibitor combination in anti‐MDA5 dermatomyositis: No significant survival benefit but reassuring safety profile

**DOI:** 10.1111/joim.70047

**Published:** 2025-11-24

**Authors:** Valentine Pagis, Quentin Astouati, Lucas Pacoureau, Yann Nguyen, Pierre Bay, Antoine Roux, Laure Gallay, Vincent Cottin, Benjamin Terrier, Alain Meyer, Charles Cerf, Mathilde Neuville, Baptiste Hervier, Arthur Renaud, Benoit Suzon, Nicolas Schleinitz, Thomas Papo, Pascaline Priou, Arsène Mekinian, Audrey Ullmer, Erwan Oehler, Noémie Gensous, Alice Berezne, Luc De Saint Martin, Thierry Marhadour, Mickaël Martin, Amélie Servettaz, Maxime Samson, Laurent Gilardin, Pierre Charles, Juliette Woessner, Thierry Carmoi, Baptiste Dilly, Wladimir Mauhin, Nicolas Baillet, Sébastien Humbert, Benjamin Thoreau, Marine Lemaitre, Claire Le Pendu, Pierre Loiseau, Thomas Quemeneur, Elodie Blanchard, Nicol Voermans, David Launay, Hilario Nunes, Olivier Benveniste, Yurdagul Uzunhan, Yves Allenbach

**Affiliations:** ^1^ Department of Internal Medicine Pitié‐Salpêtrière University Hospital AP‐HP Sorbonne University Paris France; ^2^ Department of Internal Medicine Centre de Référence des Maladies Autoimmunes Systémiques Rares du Nord Nord‐Ouest et Méditerranée (CeRAINOM) U1286 – INFINITE—Institute for Translational Research in Inflammation Univ. Lille University Hospital Lille France; ^3^ Department of Internal Medicine Bicêtre Hospital, AP‐HP Paris‐Saclay University Le Kremlin‐Bicêtre France; ^4^ Department of Internal Medicine Beaujon Hospital, AP‐HP Paris Cité University Paris France; ^5^ Department of Intensive Care Unit Mondor Hospital, AP‐HP Créteil University Créteil France; ^6^ INSERM U955, Team “Viruses, Hepatology, Cancer” Créteil France; ^7^ Department of Pneumology Foch Hospital Suresnes France; ^8^ Department of Internal Medicine Lyon Sud Hospital, Lyon Sud University Lyon France; ^9^ Department of Pneumology Lyon Hospital, Lyon University Lyon France; ^10^ Department of Internal Medicine Cochin Hospital, AP‐HP Paris Cité University Paris France; ^11^ Department of Physiology Strasbourg University Hospital Strasbourg University Strasbourg France; ^12^ Department of Reanimation Foch Hospital Suresnes France; ^13^ Department of Internal Medicine Saint‐Louis Hospital, AP‐HP Paris Cité University Paris France; ^14^ Department of Internal Medicine Nantes University Hospital Nantes France; ^15^ Department of Internal Medicine Martinique University Hospital Fort‐de‐France Martinique France; ^16^ Department of Internal Medicine La Timone University Hospital Aix‐Marseille University Marseille France; ^17^ Department of Internal Medicine Bichat Hospital, AP‐HP Paris Cité University Paris France; ^18^ Department of Pneumology Angers University Hospital Angers France; ^19^ Department of Internal Medicine Saint Antoine University Hospital, AP‐HP Sorbonne University Paris France; ^20^ Department of Internal Medicine René‐Dubois Hospital Pontoise France; ^21^ Department of Internal Medicine French Polynesia Hospital Center, Pirae Tahiti French Polynesia; ^22^ Department of Internal Medicine Bordeaux University Hospital Bordeaux France; ^23^ Department of Internal Medicine Annecy‐Genevois Hospital Annecy France; ^24^ Department of Internal Medicine Brest University Hospital Brest France; ^25^ Department of Rheumatology Brest University Hospital Brest France; ^26^ Department of Internal Medicine Poitiers University Hospital Poitiers France; ^27^ Department of Internal Medicine Robert Debré Hospital Reims France; ^28^ Department of Internal Medicine Dijon University Hospital Dijon University Dijon France; ^29^ Department of Internal Medicine Jean Verdier Hospital, AP‐HP Bondy France; ^30^ Department of Internal Medicine Institut Mutualiste Montsouris Hospital Paris France; ^31^ Department of Internal Medicine Avignon Hospital Avignon France; ^32^ Department of Internal Medicine American Hospital of Paris Paris France; ^33^ Department of Internal Medicine Elbeuf‐Louviers‐Val de Reuil Hospital Saint‐Aubin‐lès‐Elbeuf France; ^34^ Department of Internal Medicine Diaconesses Croix‐Saint Simon Hospital Paris France; ^35^ Department of Internal Medicine Basse‐Terre Hospital, Basse‐Terre Guadeloupe France; ^36^ Department of Internal Medicine Besancon University Hospital Besancon University Besancon France; ^37^ Department of Internal Medicine Tours University Hospital Tours University Tours France; ^38^ Department of Internal Medicine Foch Hospital Suresnes France; ^39^ Department of Internal Medicine Argenteuil Hospital Argenteuil France; ^40^ Department of Internal Medicine Amiens University Hospital Amiens France; ^41^ Department of Internal Medicine Valenciennes Hospital Valenciennes France; ^42^ Department of Pneumology Bordeaux University Hospital Bordeaux France; ^43^ Department of Neurology Radboud University Nijmegen Medical Centre Nijmegen the Netherlands; ^44^ Reference center for rare pulmonary diseases. Department of Pneumology Avicenne Hospital, AP‐HP Sorbonne Paris Nord University Paris France

**Keywords:** anti‐MDA5, calcineurin inhibitors, case–control study, dermatomyositis, interstitial lung disease, JAK inhibitors

## Abstract

**Objectives:**

Anti‐MDA5 dermatomyositis (anti‐MDA5 DM) is the most severe subtype of dermatomyositis, due to its pulmonary involvement. Current treatment involves corticosteroids and immunosuppressants, but variability in responses exists. This study aims to evaluate the efficacy and safety of Janus kinase (JAK)– and calcineurin–inhibitor combination (JAK–CNI) in anti‐MDA5 DM patients.

**Methods:**

A nested case–control study was conducted within a retrospective cohort of 234 anti‐MDA5 DM patients. Patients receiving JAK–CNI were matched 1:2 with comparators. All‐cause mortality or transplant within a year was compared using Cox proportional hazards models. Infectious and noninfectious side effects were also assessed.

**Results:**

Twenty‐seven patients receiving JAK–CNI were compared to 52 matched controls. Almost all these patients had pulmonary involvement. Thirty‐nine (49%) died or were transplanted during follow‐up. No significant improvement in survival or transplant‐free survival was observed with JAK–CNI compared with comparators (hazard ratios 1.02, 95% confidence intervals [0.48–2.16]). Results were consistent regardless of intensive care unit (ICU) admission status and when analyses were restricted to patients with rapidly progressive interstitial lung disease. A trend toward a beneficial effect of the JAK–CNI combination was observed in non‐ICU patients. Infectious complications were frequent (*n* = 49, 62%), with no excess risk in patients receiving JAK–CNI.

**Conclusion:**

JAK–CNI showed a similar outcome to other immunosuppressive combinations. However, as the study included the most severe cases, the potential benefit of early JAK–CNI introduction in less severe forms cannot be dismissed, as suggested by the nonsignificant trend in non‐ICU patients. Future studies are needed to clarify the optimal timing and patient selection for JAK–CNI therapy in anti‐MDA5 DM.

## Graphical abstract

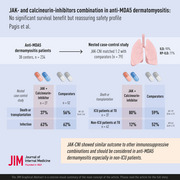

## Introduction

Respiratory involvement in systemic autoimmune diseases represents one of the most serious complications that can significantly impact prognosis. With over 40% of associated interstitial lung disease (ILD), inflammatory myopathies rank among autoimmune conditions with the highest rates of pulmonary involvement [1, 2]. This pulmonary disease stands as one of the primary causes of patient mortality, contributing to the increased mortality observed in myositis patients compared to the general population [3]. The subgroup of inflammatory myopathies, anti‐melanoma differentiation‐associated gene 5 antibody‐positive dermatomyositis (anti‐MDA5 DM), is particularly associated with ILD, affecting over 80% of patients [4, 5, 6], whereas muscular signs may be secondary or even absent. The mortality rate of anti‐MDA5 DM can reach 50% at 6 months in the case of a rapidly progressive form of ILD (RP‐ILD) [7]. Moreover, anti‐MDA5 DM mortality is significantly higher compared to other inflammatory myopathies with ILD such as anti‐synthetase syndrome, with a 5‐year mortality rate around 15% among anti‐Jo1 antibody positive [8] and approximately 25% for non‐Jo1 anti‐synthetase antibodies [9]. As a result, anti‐MDA5 DM currently has one of the highest mortality rates among idiopathic inflammatory myopathies [10] (similar to cancer‐associated myositis, with a 5‐year mortality rate at 44% [11], and compared to other systemic autoimmune disorders [11, 12, 13]).

The data regarding the treatment of this rare disease are scarce despite the significant stakes involved. Only one multicenter prospective study (non‐randomized) shows that combining high‐dose glucocorticoids, tacrolimus, and intravenous cyclophosphamide upfront (triple therapy) could be more effective than a step‐up treatment strategy, with a 6‐month survival rate higher in the triple therapy group than in the historical control group (89% vs. 33%, *p* < 0.0001) [14]. Combinations of immunosuppressants are now widely used in anti‐MDA5 DM patients, with the triple therapy being the most commonly used option in Asia and recommended by the American College of Rheumatology guidelines [15].

Despite this triple therapy, there are still refractory cases, and this combination is associated with a higher risk of infections and notably of cytomegalovirus (CMV) reactivations. Besides, a Japanese cohort study demonstrates no survival benefit of initial triple therapy over bitherapy or monotherapy on different anti‐MDA5 DM clusters, warranting the evaluation of alternative therapeutic strategies [16].

Anti‐MDA5 DM is a Type 1 interferon (IFN)‐driven autoimmune disease associated with an upregulated IFN signature in skin, muscle, and blood [17, 18, 19, 20]. Therefore, blocking IFN alpha pathway through inhibition of the downstream Janus kinase (JAK) molecules appears to be a promising strategy [21]. Case reports and series have suggested the potential benefit of JAK inhibitors [22], including for patients with ILD who do not respond to triple therapy [23]. An open‐label prospective trial showed a significantly higher 6‐month survival rate in early‐stage anti‐MDA5 DM patients with ILD receiving tofacitinib (*n* = 18) compared to a historical group (*n* = 32) [24].

There is an urgent need for data to establish new therapeutic approaches in anti‐MDA5 DM. Here, we report the results of a multicentric retrospective cohort study evaluating the efficacy and safety of a combined JAK and calcineurin–inhibitor treatment strategy in anti‐MDA5 DM patients.

## Methods

### Study design and data collection

We conducted a multicenter retrospective cohort study within the French myositis network, the Orpha Lung network, and a collaborating center in the Netherlands.

Participants were enrolled if (i) they were diagnosed with dermatomyositis according to 2018 European Neuromuscular Center criteria [23], or presented myositis and/or arthralgia and/or ILD without other etiology and (ii) with anti‐MDA5 autoantibody positivity. Anti‐MDA5 autoantibody detection was performed using line immunoassays (Euroimmun or D‐Tek). Medical records were reviewed by specialized physicians (V.P., Q.A., P.B.) to fill in a standardized data collection form. Data collection included epidemiological and clinical characteristics, laboratory results, chest high‐resolution computed tomography (HRCT), pulmonary function test results, and treatment regimens (treatment initiation date). Data collection of the cohort was updated during follow‐up and included date and status (alive, dead, or transplanted) at the last follow‐up.

### Registrations and patient consents

Patients were either part of the MASC (myositis, DNA, serum, cells) project (NCT 04637672) or included in the database registered with the *Commission Nationale de l'Informatique et des Libertés* (no. 2223905) and approved by the Institutional Review Board of the French Society for Respiratory Medicine (reference: CEPRO 2020‐067). In accordance with French and Netherlands legislation, formal written informed consent was not required for this type of study because the data studied were entirely retrospectively collected.

### Definitions and populations

For our analysis, we used two different populations, described in the statistical analysis. The first consisted of a matched population of patients receiving both a calcineurin inhibitor (CNI; tacrolimus or ciclosporin) and a JAK inhibitor (tofacitinib, baricitinib, or ruxolitinib) (JAK–CNI group) and matched comparators who did not receive this combination, regardless of treatment regimen composition (Table S2). To ensure comparability between the combination therapy group (JAK and calcineurin–inhibitor combination [JAK–CNI]) and standard treatment group (comparator group), each patient receiving both a CNI and a JAK inhibitor was matched to two patients not receiving this combination. The matching approach used a 1:2 nearest‐neighbor algorithm (MatchIt package in R) based on age at treatment initiation, number of prior treatment lines, and exact matching on sex and RP‐ILD status. This method identifies controls that minimize the Euclidean distance between cases and controls on scaled continuous variables (age at treatment initiation and number of prior treatment lines), while performing exact matching on categorical variables (sex and RP‐ILD occurrence). This approach balances both patient‐level factors, such as age and disease severity, and key clinical features, despite limitations in exact temporal matching. Balance was assessed by standardized mean differences (SMDs), with acceptable matching quality defined by SMDs below 0.2, acknowledging data constraints that precluded tighter thresholds. This compromise allowed us to retain an adequate number of matched controls while balancing important baseline characteristics. Due to data constraints, exact 1:2 matching was not fully achieved; we ultimately identified 52 matched controls (instead of the theoretical 54).

As RP‐ILD was predominantly observed at the time of diagnosis onset, instances where the date of RP‐ILD was not available were imputed as prevalent cases of RP‐ILD. To consider the line of treatment on which cases were receiving the combination therapy, matching was also based on the number of previous therapeutic lines.

ILD was defined based on HRCT imaging [25]. CT pattern was defined by the expert radiologist from each participating center as non‐specific interstitial pneumonia (NSIP), organizing pneumonia (OP), a combination of both NSIP and OP, or undetermined.

RP‐ILD was defined as an acute onset and rapid worsening of dyspnea and chest HRCT progression within 1 month or deterioration leading to respiratory failure within 3 months from the initial respiratory symptom onset [26, 27].

Prevalent RP‐ILD was defined as RP‐ILD diagnosed at baseline evaluation.

### Outcomes

The primary outcome was a composite of the occurrence of one of the two following events: death (all‐cause) or bilateral lung transplant due to anti‐MDA5 DM, whichever occurred first.

The secondary outcome of the study was a composite of the occurrence of one of the following events: death due to disease activity (ILD or DM) or transplant, whichever occurred first. Additionally, the analysis included the examination of adverse events induced by therapeutic regimens.

### Statistical analysis

In the main analysis, patients receiving JAK–CNI were compared to the comparator group described earlier. Comparison of variables between groups was performed using the Wilcoxon test and chi‐squared or Fisher's exact tests as appropriate. The chi‐squared test was applied when all expected cell counts were sufficiently large (≥5), in accordance with standard statistical guidelines. When this assumption was not met due to small sample sizes or low expected counts, we used Fisher's exact test to ensure the validity of the results.

Patients with a missing treatment initiation date or follow‐up date were excluded.

Survival and transplant‐free survival were estimated using the Kaplan–Meier method for visualization. Differences in event risk between groups were assessed using univariable Cox proportional hazards models. Patients contributed person‐time from time zero (*T*0) (e.g., the date of initiation of the JAK–CNI therapy or the corresponding *n*th therapeutic line in the matched comparator group) until the occurrence of the primary outcome, the date of last visit, or loss to follow‐up, whichever occurred first. These models provided hazard ratios (HR) with 95% confidence intervals (CI) to quantify associations between exposures and outcomes (death or transplantation). The proportional hazards assumption was evaluated using Schoenfeld residuals.

Two sensitivity analyses were performed. The first evaluated the primary outcome, stratified by whether cases and their matched comparators were hospitalized in the intensive care unit (ICU) at *T*0, to account for baseline severity.

The second focused on patients with RP‐ILD in the overall population (i.e., before matching) to assess outcomes in the most severe forms of anti‐MDA5 DM. Baseline characteristics of this subgroup are presented in Table S3.

Variables with more than 10% missing data were excluded from the analyses. For variables with ≤10% missingness, multiple imputations by chained equations were used [28]. Imputation models were selected according to variable type: predictive mean matching for continuous variables, logistic regression for binary variables, and polytomous logistic regression for categorical variables. The predictor matrix included all other variables as predictors. Variables were appropriately coded as factors or numeric. Interaction and non‐linear terms were not included. Convergence and mixing were assessed through trace plots. Ten imputed datasets were generated using 50 iterations each. Finally, we conducted sensitivity analyses by repeating the primary analyses on the complete‐case dataset (data not shown).

Descriptive analyses were presented as count (percent) for categorical variables and median [interquartile range] for continuous variables. A *p*‐value of less than 0.05 was considered statistically significant. All analyses were performed with R version 4.2.1 (R Foundation for Statistical Computing).

## Results

### Study Population

A total of 234 patients from 38 centers were initially screened. Twenty‐two were excluded before matching because of missing data. Forty‐seven received JAK inhibitors, either alone (9) or in combination (38). Among them, 27 received JAK inhibitors in combination with CNIs (JAK–CNI group) and were compared to 52 matched controls (Fig. S1).

Regarding JAK inhibitors, the majority of patients (*n* = 19) received tofacitinib at doses ranging from 10 to 30 mg daily (10 mg: *n* = 4; 20 mg: *n* = 8; 30 mg: *n* = 7). Baricitinib was used in two patients, both at 4 mg daily. Ruxolitinib was administered to six patients: 20 mg daily in two cases and 40 mg daily in four. As for CNIs, most patients (*n* = 24) received tacrolimus, whereas five received cyclosporin. Two patients were treated with both agents at different time points. Doses of CNI were adjusted according to therapeutic drug monitoring and residual levels.

Their baseline characteristics are described in Table 1. Before matching, a few statistically significant differences were observed between groups (Table S1); however, after matching, there were no clinically relevant differences in baseline characteristics between cases and controls, except for myalgias and arthralgias, which do not impact the prognosis of the disease. Patients were in their fifties at disease onset and were mostly women. Half had fever, and one third had skin ulcers and arthritis. ILD was reported in more than 90% of patients.

**Table 1:** Baseline characteristics according to treatment regimen.

	Overall (*N* = 79)	JAK–CNI (*N* = 27)	Comparator group (*N* = 52)
Age at symptom onset, years, median (IQR)	54 (45, 62)	52 (44, 59)	51 (43, 61)
Sex (female)	55 (70)	19 (70)	36 (69)
General health deterioration	74 (94)	24 (89)	50 (96)
Fever	42 (53)	11 (41)	31 (60)
ILD	71 (90)	26 (96)	45 (87)
RP‐ILD	61 (77)	21 (78)	40 (77)
Prevalent RP‐ILD	53 (65)	20 (74)	33 (63)
Dyspnea (NYHA)			
0–1	11 (14)	1 (3.7)	10 (19)
2	20 (25)	9 (33)	11 (21)
3–4	48 (61)	17 (63)	31 (60)
ILD CT pattern			
Undetermined	6 (7.6)	0 (0)	6 (12)
NSIP	30 (38)	8 (30)	22 (42)
NSIP and OP	18 (23)	11 (41)	7 (13)
OP	17 (22)	7 (26)	10 (19)
Skin lesions	66 (84)	25 (93)	41 (79)
Raynaud phenomenon	12 (15)	5 (19)	7 (13)
Skin ulcers	24 (30)	11 (41)	13 (25)
Mechanic's hands	19 (24)	8 (30)	11 (21)
Arthralgia	37 (47)	19 (70)	18 (35)
Arthritis	24 (30)	11 (41)	13 (25)
Muscular manifestation	45 (57)	16 (59)	29 (56)
Muscle weakness	22 (28)	10 (37)	12 (23)
Myalgia	28 (35)	13 (48)	15 (29)
Increased CK level	27 (34)	9 (33)	18 (35)
Cardiac involvement	4 (5.1)	4 (15)	0 (0)
Antinuclear antibody	42 (53)	15 (56)	27 (52)
Anti‐Ro52	27 (34)	11 (41)	16 (31)
Malignancy	5 (6.3)	2 (7.4)	3 (5.8)
Intensive care unit at treatment initiation	37 (47)	10 (37)	27 (52)
Number of therapeutic lines			
1	12 (15)	4 (15)	8 (15)
2	21 (27)	7 (26)	14 (27)
3	31 (39)	11 (41)	20 (38)
≥4	15 (19)	5 (19)	10 (19)
Treatments received (at any times)			
Cyclophosphamide	47 (59)	12 (44)	35 (67)
Rituximab	13 (16)	1 (4)	12 (23)
PLEX	27 (34)	11 (41)	16 (31)
ECMO support	15 (19)	6 (22)	9 (17)
Death or transplantation during follow‐up	39 (49)	10 (37)	29 (56)

*Note*: All values are expressed as numbers (percentages), unless otherwise specified.

Abbreviations: CK, creatinine kinase; CNI, calcineurin inhibitors; CT, computed tomography; ECMO, extracorporeal membrane oxygenation; ILD, interstitial lung disease; JAK, Janus kinase inhibitors; NSIP, non‐specific interstitial pneumonia; NYHA, New York Heart Association; OP, organizing pneumonia; PLEX, plasma exchange; RP‐ILD, rapidly progressive interstitial lung disease.

The prevalence of RP‐ILD at *T*0 was similar between both groups (74%; *n* = 20/27 vs. 63%; *n* = 33/52). The proportion of patients hospitalized in ICU at *T*0 was similar in both groups (37%; *n *= 10/27 vs. 52%; 27/52).

### Primary outcome

Overall, death (35/79) or lung transplant (4/79) occurred in 49% of patients (39/79) during follow‐up, including 28 events in the first year after *T*0. Of these, 37% occurred in the JAK–CNI group (10/27) and 56% in the comparator group (29/52).

The cause of death was related to disease activity in 69% (*n* = 24/35), to infection in 23% (*n* = 8/35), to cancer (*n* = 1), and to heart failure (*n *= 1). Cause of death was missing for one patient.

HRs for the incidence of the composite outcome based on the use of JAK–CNI therapy are presented in Table 2.

**Table 2:** Hazard ratios (95% confidence interval) for death (all‐cause) and transplant in the main population and according to intensive care unit hospitalization at baseline.

	Treatment regimen	Death or transplant	Hazard ratio
**Studied patients** (*N* = 79)	Comparator 52 (66) JAK–CNI 27 (34)	29 (56) 10 (37)	1.02 (0.48–2.16)
**Intensive care patients at *T*0** (*N* = 37)	Comparator 27 (73) JAK–CNI 10 (27)	16 (59) 8 (80)	1.99 (0.84–4.71)
**Nonintensive care patients at *T*0** (*N* = 42)	Comparator 25 (60) JAK–CNI 17 (40)	13 (52) 2 (12)	0.45 (0.09–2.17)

*Note*: Results are expressed as number (percentage). *T*0: date of JAK–CNI initiation or control treatment.

Abbreviations: CNI, calcineurin inhibitors; JAK, Janus kinase inhibitors.

In univariate analysis, JAK–CNI regimen was not significantly associated with a lower risk of death or lung transplant (HR 1.02, 95% CI [0.48–2.16]).

**Fig. 1:**
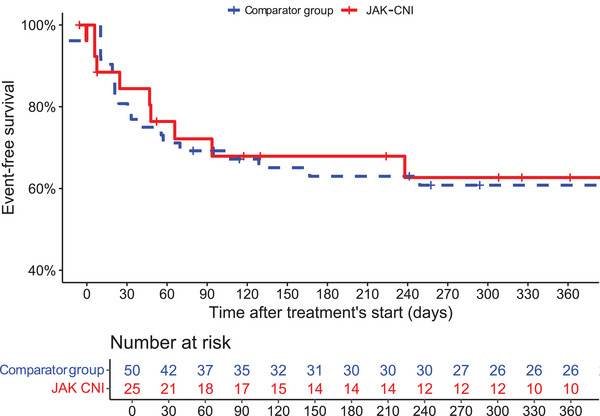
Kaplan–Meier curves for 1‐year survival and transplant‐free survival (primary outcome) according to treatment regimen. CNI, calcineurin inhibitors; JAK, Janus kinase inhibitors.

Kaplan–Meier survival curves were generated to visualize 1‐year survival and transplant‐free survival between the two groups (Fig. 1).

It should be noted that extracorporeal membrane oxygenation (ECMO) was used in a small subset of patients with RP‐ILD. Specifically, among patients who received the JAK–CNI combination therapy, six patients underwent ECMO. Among the comparator group, nine patients received ECMO. Among the nine patients in the comparator group who received ECMO, all nine died or required lung transplants. In contrast, among the six case patients who received ECMO, four died or were transplanted, whereas two survived without transplant.

When patients were hospitalized in ICU at *T*0 (*n* = 37), death or lung transplant was recorded in 80% (*n* = 8/10) and 59% (*n* = 16/27) (entire follow‐up), with no difference between JAK–CNI group and comparator group (Table 2). Among patients not hospitalized in ICU at *T*0 (*n* = 42), death or lung transplant occurred more frequently in the comparator group (52%, *n* = 13/25, versus 12%, *n* = 2/17); nevertheless, this difference was not significant (Table 2).

In the subgroup of patients with RP‐ILD (*n* = 73), death or lung transplant occurred in 48% (*n* = 10/21) in the JAK–CNI group versus 62% (*n* = 32/52) in the comparator group with an HR of 0.96, 95% CI [0.46–2.0] (Table 3).

**Table 3:** Hazard ratios (95% confidence interval) for death (all‐cause) and transplant in the rapidly progressive interstitial lung disease (RP‐ILD) population (not matched).

	Treatment regimen	Death or transplant	Hazard ratio
**Studied patients** (*N* = 73)	Comparator 52 (71) JAK–CNI 21 (29)	32 (62) 10 (48)	0.96 (0.46–2.0)

*Note*: Results are expressed as number (percentage). *T*0: date of JAK–CNI initiation or control treatment.

Abbreviation: CNI, calcineurin‐inhibitors; JAK, Janus kinase inhibitors.

### Secondary outcomes

In the studied population (*n* = 79), 30% of patients (*n* = 24) died from disease activity (ILD or DM) or had a transplant, with, respectively, 21% among the JAK–CNI group and 79% among those in the comparator group.

**Fig. 2:**
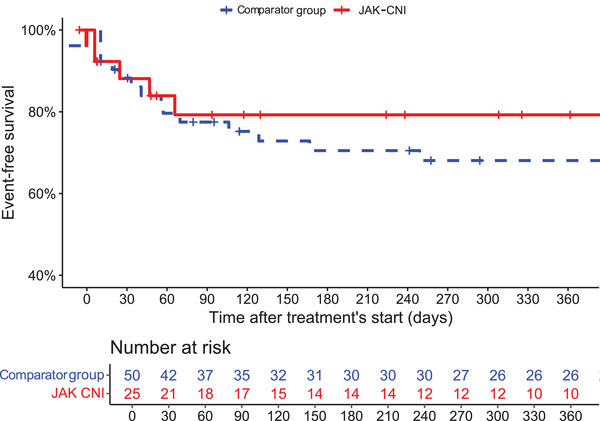
Kaplan–Meier curves for the risk of the secondary outcome with specific mortality according to treatment regimen. CNI, calcineurin inhibitors; JAK, Janus kinase inhibitors.

In univariate analysis, JAK–CNI therapy regimen was not significantly associated with a lower risk of the composite secondary outcome (HR 0.84, 95% [0.33–2.16]) (Fig. 2).

### Safety analysis

Adverse events are presented in Table 4. Infections occurred in more than half of all patients (62%, *n* = 49/79). There were no significant differences regarding the total number of infections that occurred in patients in both groups, nor the number of pneumocystis pneumonia nor viral infections (VZV/HSV or CMV infection). Of the 79 patients, 75% were taking anti‐infective prophylaxis (anti‐pneumocystis and/or anti‐VZV), with no difference between JAK–CNI group and the comparator group.

**Table 4:** Infectious complications and other adverse events.

	Overall (*N* = 79)	JAK–CNI (*N* = 27)	Comparator group (*N* = 52)	*p*‐Value
Infection (total)	49 (62)	17 (63)	32 (62)	0.98
Bacterial pneumonitis	29 (37)	10 (37)	19 (37)	0.88
Viral infection	26 (33)	7 (26)	19 (37)	0.36
Fungal infection	14 (18)	4 (15)	10 (19)	0.73
Pneumocystis pneumonia	8 (10)	3 (11)	5 (10)	>0.99
*Aspergillus*	2 (3)	2 (7)	0 (0)	0.15
Urinary infection	7 (9)	2 (7)	5 (10)	0.70
Other infections	11 (14)	6 (22)	5 (10)	0.17
Infectious prophylaxis	59 (75)	23 (85)	36 (69)	0.24
Other adverse events^a^	13 (16)	6 (22)	7 (13)	0.35

*Note*: Results are expressed as number (percentage). Pearson's chi‐squared test; Fisher's exact test; results are considered significant if the *p*‐value is less than 0.05.

^a^ Acute renal failure, cytolysis, EBV‐induced hemopathy, femoral head necrosis, mania episode, pancytopenia, pulmonary embolism, thrombotic microangiopathy.

## Discussion

Although anti‐MDA5 DM–associated mortality remains excessively high, the existing literature regarding overall survival is scarce and limited by population size and heterogeneity. To the best of our knowledge, this represents the largest multicenter observational cohort in Western countries, providing clinically pragmatic and relevant endpoints (mortality or transplant).

In this retrospective cohort study, the use of JAK–CNI combination therapy was not associated with a statistically significant improvement in overall survival or transplant‐free survival when compared to other immunosuppressive regimens in the matched population.

We decided to include all patients who received the JAK–CNI combination, regardless of baseline pulmonary involvement, in order to capture the full spectrum of anti‐MDA5 DM. ILD is not invariably present at disease onset, and severity may manifest later in the clinical course. This strategy reflects real‐world practice, particularly in Caucasian populations where, apart from RP‐ILD, no robust prognostic risk factors have yet been clearly defined.

Although the initial study cohort of 214 patients exhibited the classical phenotype of the disease (80% ILD, 68% myositis), with 23% of patients either dying or requiring lung transplant during follow‐up, the JAK–CNI matched population represented an even more severe population, with 90% of patients having ILD and 49% dying or requiring transplant (Table S1). This imbalance in disease severity may have biased the analysis toward non‐significant results, as JAK–CNI therapy was preferentially administered to the most severe cluster of patients according to the literature (80% 3‐month mortality rate) [6]. To ensure that this imbalance did not drive our findings, we performed a sensitivity analysis restricted to patients with RP‐ILD (unmatched population), where results remained consistent with the main analysis.

Interestingly, in non‐ICU patients, although the primary outcome did not reach statistical significance, the HR was 0.45 [0.09–2.17], suggesting a trend toward a beneficial effect of the JAK–CNI combination, which might have reached significance in a larger cohort. Therefore, we cannot formally exclude a beneficial effect of an early introduction of JAK–CNI in a less severe subset of the population.

Among the most severe patients, only a small proportion required ECMO. Among the nine patients in the comparator group who required ECMO, all either died or underwent lung transplantation. Of note, in the JAK–CNI group, among the six patients in who required ECMO, two survived without transplantation, including one with concomitant infectious pneumonia. The potential benefit of ECMO should be interpreted with caution due to the small sample size and the lack of standardized indications across centers. Previous data suggest that ECMO is primarily used as a bridge to transplantation rather than as a bridge to recovery in anti‐MDA5 DM [29].

Although current therapeutic guidelines in anti‐MDA5 DM are largely empirical, CNIs in association with corticosteroids are routinely used [30]. A prospective non‐randomized Japanese study showed that the 6‐month survival rate was higher in the triple therapy group (high‐dose corticosteroids, CNIs, and intravenous cyclophosphamide) than in the historical comparator group (89% vs. 33%, *p* < 0.0001) [14]. However, the benefit of such a regimen is counterbalanced with an increased infectious risk [14, 31].

Because activation of the Type I IFN signaling pathway is thought to play a key role in anti‐MDA5 DM [20], JAK inhibitors may constitute an alternative strategy. Along that line, preliminary case series and small prospective open‐label studies showed promising results as reviewed elsewhere [32], although these preliminary reports often lacked an appropriate control population. An open‐labeled (before–after) study evaluated the combination of tofacitinib with steroids as a first‐line regimen in 18 patients (compared to 32 historical controls) and showed very promising data with a significantly higher 6‐month survival rate (100% vs. 78%, *p* = 0.04) [24]. These exceptional results were reported on a subset of patients with mild respiratory severity (forced vital capacity >50%). Our study does not confirm such efficacy in every patient. However, severity comparison between cohorts is limited by the non‐availability of respiratory function tests in our.

Moreover, a regimen including CNIs with tofacitinib was evaluated against CNIs only in a small retrospective cohort (14 and 22 patients, respectively) and displayed a significantly higher 6‐month survival rate (>90% vs. >60%, *p* = 0.045) [20], when the 6‐month survival rate was 70% in the JAK–CNI group in our study. However, no matching strategy was used between the evaluated groups, nor did the authors report the incidence of ILD/RP‐ILD, preventing the conclusion of a true survival improvement by JAK–CNI combination. In a recent multicenter retrospective study, tofacitinib was found to reduce the mortality of newly diagnosed anti‐MDA5 DM with ILD by 28% compared to CNI. The benefit of tofacitinib over CNI was observed in younger patients without RP‐ILD, particularly when used without additional immunosuppressants (probably reflecting a less severe subset of the population) [33]. The results suggest the benefit from the early addition of a JAK inhibitor in patients with less severe disease. This is consistent with our findings, which show a trend—although not statistically significant—toward the introduction of JAK–CNI in the subgroup of patients not admitted to ICU at treatment introduction. However, in this study, patients treated with the JAK–CNI combination were excluded, precluding a direct comparison with our study. On the other hand, JAK inhibitors have been reported in a small case series to be beneficial as rescue therapy for refractory ILD combined with other poor prognosis factors (survival rate 60%, 3/5 vs. 0/6 among historical controls) [23].

Based on our findings, these previous studies may have been affected by the lack of suitable controls to account for disease severity. Additionally, there could also be a publication bias resulting in the overestimation of JAK inhibitors’ benefit [32]. Our findings provide some nuance to the existing literature. Although the benefit of JAK inhibitors in combination with CNI is likely to be of a lesser magnitude than previously reported, one cannot exclude the possibility of a yet‐to‐be‐defined subset of patients that could derive benefits from such combination therapy. A prospective study is needed to assess the efficacy of such combination therapy, and considering the heterogeneity between the Asian and non‐Asian populations, a prospective study conducted in Western countries is also necessary. In our study, most of the JAK–CNI patients received tofacitinib (*n* = 19) at doses higher than 10 mg per day, in line with recent reports suggesting a benefit of dose escalation to 20 mg per day in refractory cases [34].

Beyond efficacy, we must consider the toxicity and, in particular, the infectious risk associated with immunosuppressive combinations. JAK inhibitors, by blocking IFN pathway (a major actor in antiviral immunity) [35], are a risk factor for viral infections such as zoster infections [36]. Viral infections in anti‐MDA5 DM are a major concern since CMV reactivations were reported as high as 64% in anti‐MDA5 DM patients and were associated with cyclophosphamide, CNI, or a combination of both in an observational retrospective study [31]. CMV reactivation was reported in up to 100% (5/5) of patients who received tofacitinib in addition to triple therapy [23]. A recent review of 30 anti‐MDA DM patients with ILD treated with tofacitinib reported infectious adverse events in 30% of cases, not only viral infections (20% CMV reactivations, 10% VZV‐associated diseases) but also bacterial pneumonias (17%) and fungal infections (13%) [32]. Most infections reported are respiratory, suggesting local immunosuppression related to the disease itself [37].

In our study, we also reported a high frequency of infectious complications, but the JAK–CNI combination therapy was not associated with an increased risk. This observation holds true even when specifically examining viral infections or pneumocystis pneumonias. This observation must be tempered with the fact that most patients received infectious prophylaxis.

Our study is subject to several limitations. First, as a retrospective study, we cannot exclude residual bias and unmeasured confounders. To partially address this concern, we employed a matching process to account for known prognostic factors at initial presentation. Due to the retrospective nature of the study and missing data, some prognostic markers (e.g., ferritin levels, anti‐MDA5 antibody titers, and CT severity scores) could not be included in the matching process. However, these prognostic factors may have a mild individual impact on the outcome and have not been validated in Western populations. Moreover, due to the more recent introduction of JAK inhibitors, whereas the comparator group includes patients treated before the availability of JAK inhibitors, it was difficult to perform matching based on treatment initiation date. This may have introduced a temporal bias, as clinical practices may have evolved over time. The small sample size receiving the JAK–CNI combination, inherent to the rarity of this disease, restricts our ability to draw definite conclusions.

Finally, the heterogeneity of immunosuppressive regimens in the comparator group (Table S2) is also a limitation. Although all patients in the comparator group were selected for not having received JAK inhibitors, they could have been treated with CNI and other immunosuppressive agents. This variability reflects real‐world treatment practices and the absence of a standardized therapeutic approach across centers. However, it may introduce confounding and limit the direct comparability between groups. We attempted to mitigate this through our matching strategy, but residual confounding cannot be excluded.

In conclusion, in rare and severe diseases like anti‐MDA5 DM, with no available randomized controlled studies, retrospective studies provide useful data to guide therapeutic decisions. Our results moderate the first promising results about JAK inhibitor use and emphasize the need for an early initiation and prospective monitoring of the treatment effect of intensive immunosuppressive treatment, regardless of the molecule. Both the combination of JAK–CNI and the triple therapy (widely recognized as the conventional treatment in most Asian studies) remain viable options for the treatment of anti‐MDA5 DM.

Future studies should aim to decipher the heterogeneity of the disease to provide more accurate prognostic information for early identification of patients who would benefit from prompt initiation of aggressive therapies.

## Conflict of interest statement

The authors declare no conflicts of interest.

## Funding information

No specific funding was received from any bodies in the public, commercial, or not‐for‐profit sectors to carry out the work described in this article.

## Supporting information




**Fig. S1**: Flow chart. CNI: calcineurin inhibitors; JAK: Janus kinase inhibitors; anti‐MDA5 DM: anti–melanoma differentiation‐associated gene 5 antibody positive dermatomyositis.
**Table S1**: Clinical characteristics of the population before matching.
**Table S2**: Detailed treatment regimen for the comparator group.
**Table S3**: Clinical characteristics of the RP‐ILD population without matching.

## Data Availability

Data are available on reasonable request.
