# Supplementary material for: Janus kinase and calcineurin‐inhibitor combination in anti‐MDA5 dermatomyositis: No significant survival benefit but reassuring safety profile
Source: J Intern Med. 2025 Nov 24;299(2):228–40. doi: 10.1111/joim.70047 (PMC12789280; doi:10.1111/joim.70047)
Supplement: Supplementary file 1 — Fig. S1: Flow chart. CNI: calcineurin inhibitors; JAK: Janus kinase inhibitors; anti‐MDA5 DM: anti–melanoma differentiation‐associated gene 5 antibody positive dermatomyositis. Table S1: Clinical characteristics of the population before matching. Table S2: Detailed treatment regimen for the comparator group. Table S3: Clinical characteristics of the RP‐ILD population without matching. [file JOIM-299-228-s001.docx]

**Supplementary Material**- JAK- and calcineurin-inhibitors combination in anti-MDA5 dermatomyositis: no significant survival benefit but reassuring safety profile

**
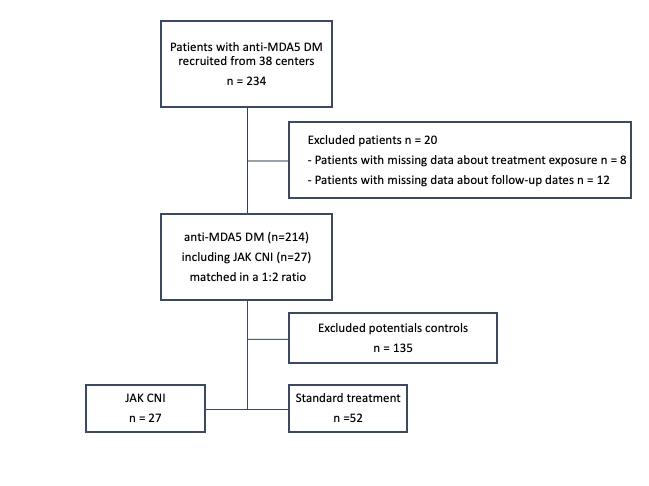
**

**Supplementary Figure S1: Flow chart.** CNI: Calcineurin-inhibitors; JAK: Janus kinase inhibitors; Anti-MDA5 DM: Anti- melanoma differentiation-associated gene 5 antibody positive dermatomyositis

**Supplementary table S1. Clinical characteristics of the population before matching**

|  | **Overall**  (n=214) | **JAK-CNI**  (n=27) | **Comparator group**  (n=185) | **p-value** |
| --- | --- | --- | --- | --- |
| **Age at symptom onset, years,** median (IQR) | 49 (36 ; 58) | 51 (44 ; 59) | 49 (36 ; 58) | 0.47 |
| **Sex (Female)** | 141 (66) | 19 (70) | 122 (65) | 0.60 |
| **General Health Deterioration** | 172 (80) | 24 (89) | 148 (80) | 0.23 |
| **Fever** | 80 (37) | 11 (41) | 69 (37) | 0.70 |
| **ILD** | 171 (80) | 26 (96) | 145 (78) | 0.023 |
| **RP-ILD** | 73 (34) | 20 (74) | 52 (28) | <0.001 |
| **Dyspnea (NYHA)** |  |  |  | <0.001 |
| 0 - 1 | 74 (35) | 1 (3.7) | 73 (39) |  |
| 2 | 71 (33) | 9 (33) | 62 (34) |  |
| 3 - 4 | 69 (32) | 17 (63) | 52 (28) |  |
| **ILD CT-Pattern** |  |  |  | 0.014 |
| Undetermined | 14 (6.5) | 0 (0) | 14 (7.5) |  |
| Normal | 39 (18) | 1 (3.7) | 38 (20) |  |
| NSIP | 78 (36) | 8 (30) | 70 (37) |  |
| NSIP OP | 48 (22) | 11 (41) | 37 (20) |  |
| OP | 35 (16) | 7 (26) | 28 (15) |  |
| **Skin lesion** | 192 (90) | 25 (93) | 167 (89) | > 0.99 |
| Raynaud phenomenon | 59 (28) | 5 (19) | 54 (29) | 0.26 |
| Skin ulcers | 83 (39) | 11 (41) | 72 (39) | 0.82 |
| Mechanic’s hands | 76 (33) | 8 (30) | 68 (36) | 0.49 |
| **Arthralgia** | 152 (71) | 19 (70) | 133 (71) | 0.94 |
| **Arthritis** | 79 (36) | 11 (41) | 68 (36) | 0.66 |
| **Anti-Ro52** | 56 (26) | 11 (37) | 45 (24) | 0.065 |
| **Antinuclear antibody** | 113 (53) | 15 (59) | 98 (52) | 0.76 |
| **Muscular manifestation** | 145 (68) | 16 (59) | 129 (69) | 0.31 |
| Muscle weakness | 60 (28) | 10 (37) | 50 (27) | 0.27 |
| Myalgia | 100 (47) | 13 (48) | 87 (47) | 0.87 |
| Elevated CK level | 62 (28) | 9 (33) | 53 (28) | 0.59 |
| **Cardiac involvement** | 18 (8.4) | 4 (15) | 14 (7.5) | 0.26 |
| **Death (all-cause) or transplantation** | 49 (23) | 10 (37) | 39 (21) | 0.066 |
| **Intensive care unit hospitalization (at any type during follow-up)** | 59 (28) | 15 (56) | 44 (24) | <0.001 |
| **Number of therapeutic lines** |  |  |  | 0.13 |
| 1 | 64 (30) | 4 (15) | 60 (31) |  |
| 2 | 61 (29) | 7 (26) | 54 (29) |  |
| 3 | 48 (22) | 11 (41) | 37 (20) |  |
| 4 | 32 (15) | 4 (15) | 28 (15) |  |
| 5 | 9 (4.2) | 1 (3.7) | 8 (4.3) |  |
| All values are expressed as number (percentage), unless otherwise specified  CK: Creatinine kinase; CT: computed tomography; ILD: Interstitial lung disease; NSIP: Non-specific interstitial pneumonia; OP: Organising pneumonia; RP-ILD: Rapidly progressive interstitial lung disease  Wilcoxon rank sum test; Pearson’s Chi-squared test; Fisher’s exact test; Results are considered significant if the p-value is less than 0.05 | | | | |

**Supplementary Table S2: Detailed treatment regimen for the comparator group**

| **Treatments received** | **1^st^ line** | **2^nd^ line** | **3^rd^ line** | **4^th^ line** |
| --- | --- | --- | --- | --- |
| **Steroids alone (or with HCQ)** | 16 | 5 | 5 | 5 |
| **Steroids + Methotrexate** | 5 | 0 | 0 | 0 |
| **Steroids + AZA** | 0 | 3 | 4 | 0 |
| **Steroids + MMF** | 6 | 5 | 1 | 1 |
| **Steroids + CNI** | 0 | 4 | 2 | 0 |
| **Steroids + Cyclophosphamide** | 13 | 8 | 12 | 0 |
| **Steroids + Rituximab** | 0 | 4 | 1 | 3 |
| **Steroids + PLEX (or IVIG)** | 0 | 1 | 0 | 3 |
| **Steroids + MMF + IVIG** | 0 | 0 | 4 | 1 |
| **Steroids + MMF or AZA + Rituximab** | 0 | 0 | 4 | 0 |
| **Steroids + CNI + PLEX (+/- IVIG)** | 4 | 6 | 0 | 2 |
| **Steroids + Cyclophosphamide + PLEX (or IVIG)** | 8 | 4 | 0 | 0 |
| **Steroids + CNI + Cyclophosphamide** | 0 | 2 | 0 | 0 |
| **Steroids + Rituximab + CNI** | 0 | 2 | 0 | 0 |
| Values are expressed as number  AZA: Azathioprine; CNI: Calcineurin-inhibitors; HCQ: Hydroxychloroquine; IVIG; Intraveinous immunoglobulins; MMF: Mycophenolate Mofetil; PLEX: Plasma exchange | | | | |

**Supplementary table S3. Clinical characteristics of the RP-ILD population without matching**

|  | **Overall**  (n=73) | **JAK-CNI**  (n=21) | **Comparator**  (n=52) | **p-value** |
| --- | --- | --- | --- | --- |
| **Age at symptom onset, years,** median (IQR) | 55 (44 ; 62) | 52 (44 ; 60) | 56 (44 ; 62) | 0.55 |
| **Sex (Female)** | 42 (58) | 14 (67) | 22 (54) | 0.32 |
| **General Health Deterioration** | 69 (95) | 20 (95) | 49 (94) | >0.99 |
| **Fever** | 44 (60) | 10 (48) | 34 (65) | 0.16 |
| **RP-ILD** | 73 (100) | 21 (100) | 52 (100) | >0.99 |
| **Dyspnea (NYHA)** |  |  |  | >0.99 |
| 0 - 1 | 2 (2.7) | 0 (0) | 2 (3.8) |  |
| 2 | 15 (21) | 4 (19) | 11 (21) |  |
| 3 - 4 | 56 (77) | 17 (81) | 39 (75) |  |
| **ILD CT-Pattern** |  |  |  | 0.66 |
| Undetermined | 5 (6.8) | 0 (0) | 5 (9.6) |  |
| Normal | 1 (1.4) | 0 (0) | 1 (1.9) |  |
| NSIP | 29 (40) | 8 (38) | 21 (40) |  |
| NSIP OP | 26 (36) | 9 (43) | 17 (33) |  |
| OP | 12 (16) | 4 (19) | 8 (15) |  |
| **Skin lesion** | 61 (84) | 19 (90) | 42 (81) | 0.49 |
| Raynaud phenomenon | 12 (16) | 4 (19) | 8 (15) | 0.73 |
| Skin ulcers | 27 (37) | 10 (48) | 17 (33) | 0.23 |
| Mechanic’s hands | 25 (34) | 5 (24) | 20 (38) | 0.23 |
| **Arthralgia** | 39 (53) | 13 (62) | 26 (50) | 0.36 |
| **Arthritis** | 21 (29) | 7 (33) | 26 (50) | 0.58 |
| **Muscular manifestation** | 44 (60) | 12 (57) | 32 (62) | 0.73 |
| Muscle weakness | 21 (29) | 7 (33) | 14 (27) | 0.58 |
| Myalgia | 27 (37) | 9 (43) | 18 (35) | 0.51 |
| Elevated CK level | 24 (33) | 7 (33) | 17 (33) | 0.96 |
| **Cardiac involvement** | 5 (5.8) | 2 (9.5) | 3 (5.8) | 0.62 |
| **Antinuclear antibody** | 36 (49) | 12 (57) | 24 (46) | 0.40 |
| **Anti-Ro52** | 26 (36) | 10 (48) | 16 (31) | 0.17 |
| **Intensive care unit hospitalization (at any type during follow-up)** | 49 (67) | 10 (48) | 39 (75) | 0.024 |
| **Number of therapeutic lines** |  |  |  | 0.012 |
| 1 | 22 (30) | 3 (14) | 19 (37) |  |
| 2 | 18 (25) | 4 (19) | 14 (27) |  |
| 3 | 16 (22) | 10 (48) | 6 (12) |  |
| 4 | 15 (21) | 3 (14) | 12 (23) |  |
| 5 | 2 (2.7) | 1 (9.5) | 1 (1.9) |  |
| All values are expressed as number (percentage), unless otherwise specified  CK: Creatinine kinase; CT: computed tomography; ILD: Interstitial lung disease; NSIP: Non-specific interstitial pneumonia; OP: Organising pneumonia; RP-ILD: Rapidly progressive interstitial lung disease  Wilcoxon rank sum test; Pearson’s Chi-squared test; Fisher’s exact test; Results are considered significant if the p-value is less than 0.05 | | | | |
